# Compressive Behavior of Al-TiB_2_ Composite Foams Fabricated under Increased Pressure

**DOI:** 10.3390/ma14175112

**Published:** 2021-09-06

**Authors:** Yang Yu, Zhuokun Cao, Jiaqi Wang, Ganfeng Tu, Yongliang Mu

**Affiliations:** School of Metallurgy, Northeastern University, Shenyang 110819, China; 1610507@stu.neu.edu.cn (Y.Y.); 1901540@stu.neu.edu.cn (J.W.); tugf@smm.neu.edu.cn (G.T.); muyl@smm.neu.edu.cn (Y.M.)

**Keywords:** in situ TiB_2_ composite foams, foaming pressure, deformation behavior, energy absorption efficiency

## Abstract

The application of increased pressure was used as a strategy to investigate the effect of different cell structures on the mechanical properties of Al-TiB_2_ composite foams. In situ Al-xTiB_2_ (x = 5, 10 wt.%) composites were foamed under three different pressures (0.1 MPa, 0.24 MPa, 0.4 MPa) through the liquid melt route. The macro-structure of the composite foams was analyzed in terms of cell size distribution measured by X-ray microcomputed tomography (micro-CT). It was found that the mean cell size decreases, and the cell size distribution range narrows with increasing pressure. Uniaxial compression tests revealed that the stress fluctuation (R_sd_) of 10TiB_2_ foams is larger than that of 5TiB_2_ foams under the same pressure. Moreover, cell size refinement causes the simultaneous deformation of multi-layer cells, which leads to an enhancement in the energy absorption efficiency and specific energy absorption. The comparison of experimental data with theoretical predictions (G&A model) is discussed.

## 1. Introduction

Metal foams are porous materials in which one phase is gaseous and the others are dense metals or alloys. Aluminum foams with a closed cell structure have high specific strength and excellent energy absorption ability and have been receiving increasing attention in various engineering fields such as construction, transportation, and aerospace [1,2,3].

The mechanical properties of aluminum foams depend on both their morphological and micro-structural features. A number of studies have been conducted to investigate the influence of morphological features (such as defects, cell shape anisotropy, cell wall thickness, etc.) on mechanical performance. Defects (curved and serrated cell walls, missing cells, partially coupled cells, etc.) could significantly reduce the plastic collapse stress of aluminum foams [4,5]. The anisotropy cell shape leads to higher plastic collapse stress and energy absorption capacity in the longitudinal direction than in the transverse direction [6]. Additionally, high degrees of local anisotropy are likely to induce collapse bands [7]. However, the effect of the significant change of cell size under increased pressure on mechanical properties has not been reported.

As is well known, the cell wall material of metal foams has, in general, significant effects on their mechanical properties. It is widely accepted that the bending and buckling of cell walls dominate deformation behavior in the plateau stage. Several model analyses and simulations have been used, which have indicated the bending, buckling, and fracture of a simplified unit cell structure [8,9,10,11,12]. The most typical one is the Gibson–Ashby model (G&A model) [8]. Moreover, the nature of the cell wall material has an effect on compressive behavior. Past studies have suggested that the alloy composite selected for foaming experiments has an effect on mechanical behavior [8,13,14,15]. Furthermore, large ex situ particle additions (such as SiC, Al_2_O_3_, and Y_2_O_3_, 6–20 μm) during the fabrication of closed-cell foams can cause the metal matrix to be brittle, which can deteriorate the mechanical properties of composite foams. Recently, in situ Al-TiB_2_ composite materials have attracted wide attention due to the in situ TiB_2_ particle micron size (<2 μm) and a good bond with the matrix compared to large ex situ particles, which can avoid deteriorating the mechanical properties [16,17,18]. However, the effect of different cell structures on the mechanical properties of in situ Al-TiB_2_ composite foams was not presented in these studies.

The purpose of this paper was to investigate the effect of different cell structures on the mechanical properties of Al-TiB_2_ composite foams. In situ Al-xTiB_2_ (x = 5, 10 wt.%) composite foams were prepared by the melt route under three different pressures. The pore structure was characterized by X-ray microcomputed tomography (micro-CT), and the micro-structure was characterized by scanning electron microscopy (SEM). The compressive performance of in situ TiB_2_ composite foams was also investigated.

## 2. Materials and Methods

### 2.1. Foam Preparation

**Figure 1:**
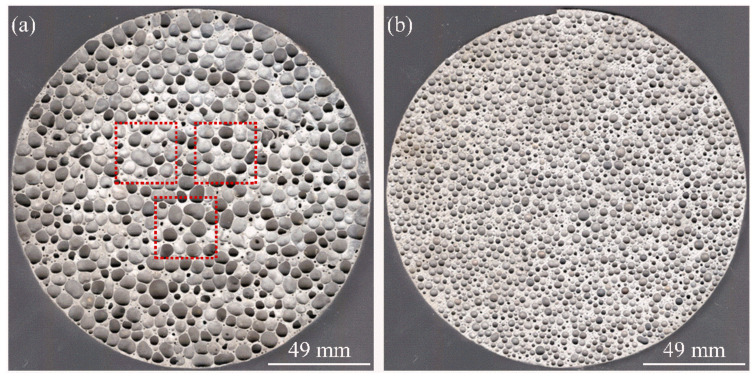
The top-view of the fabricated cylindrical sample; the dotted box is the cutting position of the compressed samples: (**a**) 10TiB_2_-0.1P; (**b**) 10TiB_2_-0.24P.

In situ Al-10TiB_2_ composite ingots were produced by Shandong Binzhou Huachuang Metal Co., Ltd. China. The chemical composition is shown in Table 1. Al-5TiB_2_ melt was obtained by adding pure aluminum (purity 99.6%) to the Al-10TiB_2_. First, the composite melt was obtained by heating 2.5 kg of composite placed in a stainless steel round crucible (inner diameter 172 mm and wall thickness 7 mm) in a furnace at 750 °C for 5 h. After the temperature decreased to 720 °C, calcium (2.25%, purity 99.9%) was added to the melt and was then stirred continuously for 5 min at 400 rpm. Then, titanium hydride powder (mean diameter: 22 μm, purity 99.4%, pre-treated at 400 °C for 30 min in air), which was to be used as foaming agent, was introduced into the melt at 690 °C. During this addition, the melt was stirred continuously for 3 min at 1200 rpm to obtain a homogeneous distribution. After this, the melt was held isothermally inside of the furnace in nitrogen at the setting pressure value. Finally, the sample was removed and was allowed to solidify by means of air cooling. The detailed procedure and setup are shown in Ref. [19]. The foaming parameters are shown in Table 2. The fabricated foams will be referred to as xTiB_2_-yP (x = 5.10; y = foaming pressure). Figure 1 shows the top view of the fabricated cylindrical sample. A total of three 30 × 30 × 30 mm^3^ specimens were cut from the samples fabricated under each pressure by a wire-cutting machine. The sampling position was the same cross-section in the middle of the cylindrical sample, as shown by the dotted box in Figure 1.

**Figure 2:**
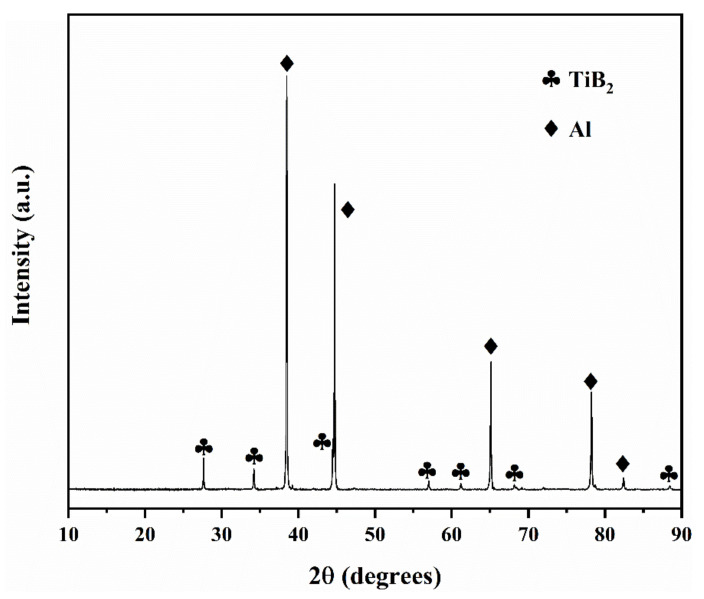
XRD pattern of Al-10TiB_2_ composite (a. u. = arbitrary unit).

Figure 2 shows the XRD pattern of the Al-10TiB_2_ composite. The characteristic Al and TiB_2_ peaks can be observed in the composite.

### 2.2. Structural and Mechanical Characterization

The density (*ρ*) measurement of the foams was conducted by dividing their weight by their volume. Relative density (*ρ*/*ρ_s_*) is defined as the ratio of the density of the foam to the density of the constituent dense solid (2.73 and 2.79 g/cc were taken as the densities of the 5 and 10TiB_2_ dense solids) [16]. Macrostructural parameters such as equivalent diameter and cell circularity were characterized using micro-CT (from Dandong Aolong Ray Instrument Group Co. Ltd., Dandong, China). The X-ray tube current and voltage were set to be 90 µA and 90 kV, respectively. The tomographic images of the samples were obtained by rotating the samples 360° in steps of 1°. After each step, radiation projections were performed, and two-dimensional slices were obtained based on the back-projection reconstruction algorithm. The gray threshold was adjusted to clearly identify the cell pores and cell walls in the CT images. Image analysis software (Image-Pro Plus 6.0) was used to measure the above-mentioned structural parameters. The mean cell diameter (*D_mean_*) was determined by the cell size distribution based on the area fraction. The lognormal distribution function fits the cell size distributions better than a Gaussian distribution function [14]. The mean circularity (*C_mean_*) of the cells was determined by calculating the arithmetic average. The reader is referred to Ref. [14] for more details regarding the explanation of the macrostructure. Microstructural analysis was conducted using a scanning electron microscope (SEM: TESCAN MIRA3, TESCAN, Brno, Czech Republic) on the cell walls of the foam before compression testing.

Quasi-static compression tests were conducted in a standard universal testing machine (CMT5105, MTS Systems, Shanghai, China) at a displacement rate of 2 mm/min up to nominal strains of 8%, 20%, and 80%. Extra micro-CT was implemented on the foams that were strained up to 8% (or 6%) and 20%. Note that these two strains approximately correspond to the beginning of the plastic deformation of the samples and to the steady-state plastic deformation regime (or the plateau stage in the stress-strain curve).

## 3. Results and Discussion

### 3.1. Foam Macro-Structural Characterization

**Figure 3:**
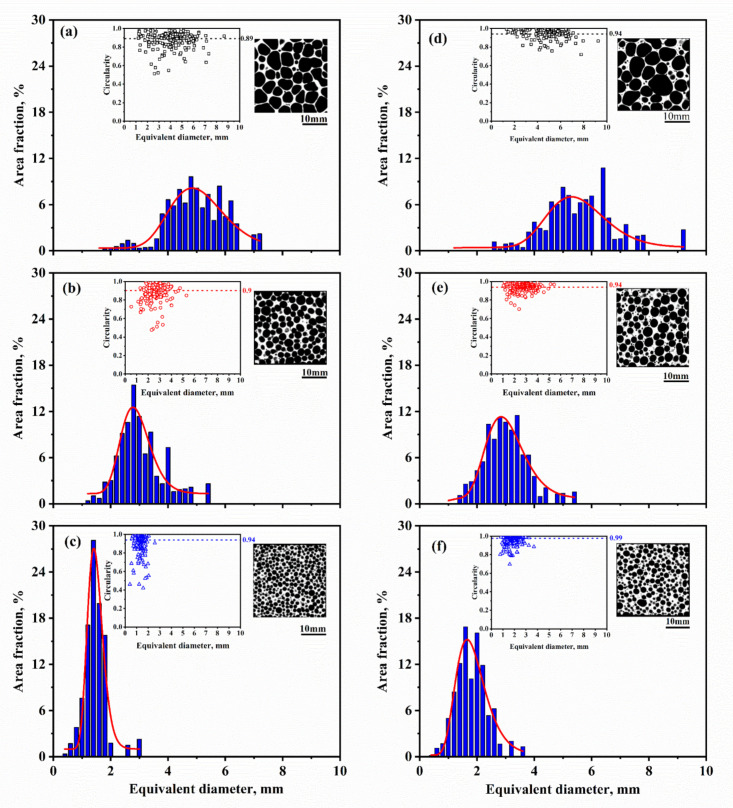
The structure and corresponding cell size distribution (red line: fitting of lognormal distribution function) and circularity of foam specimens: (**a**) 5TiB_2_-0.1P; (**b**) 5TiB_2_-0.24P; (**c**) 5TiB_2_-0.4P; (**d**) 10TiB_2_-0.1P; (**e**) 10TiB_2_-0.24P; (**f**) 10TiB_2_-0.4P.

Figure 3 shows the structure and corresponding cell size distribution and circularity of the composite foams fabricated under different pressures. When the foaming pressure increases, there is a significant decrease in the mean cell diameter and the cell size distribution range, leading to a homogeneous cell structure. The reduction of gravity drainage and bubble coalescing under increased pressure is responsible for the homogeneous cell structure [19]. On the other hand, 5TiB_2_ foams exhibit a narrower cell size distribution range compared to the 10TiB_2_ foams under the same foaming pressure. Moreover, the circularity increases with the increase of the foaming pressure. When the pressure increases from 0.1 MPa to 0.24 MPa, the circularity values of the 5TiB_2_ and 10TiB_2_ foams do not increase much (there is basically no increase). When the pressure increases to 0.4 MPa, the circularity values of the two foams increase significantly. The macrostructural characteristics of composite foams such as *ρ*, *D_mean_*, and *C_mean_* are listed in Table 3. It can be seen that the 5TiB_2_ foams exhibit smaller *ρ*, *D_mean_*, and *C_mean_* compared to the 10 TiB_2_ foams under the same foaming pressure. In the present paper, the density of the foams is related to the expansion height of the foams because the quality of the samples that have not been cut is the same. It has been reported that 5TiB_2_ composite foams have better expansion compared to 10TiB_2_ composite foams [20]. In addition, it has also been claimed that the rupture in cell walls causes bubble coalescence and that this coalescence occurs more frequently in the case of 10TiB_2_ composite foams compared to 5TiB_2_ composite foams [20], which results in the mean cell size of the 10TiB_2_ composite foams being higher than that of the 5TiB_2_ composite foams for the same foaming pressure.

### 3.2. Foam Micro-Structural Characterization

**Figure 4:**
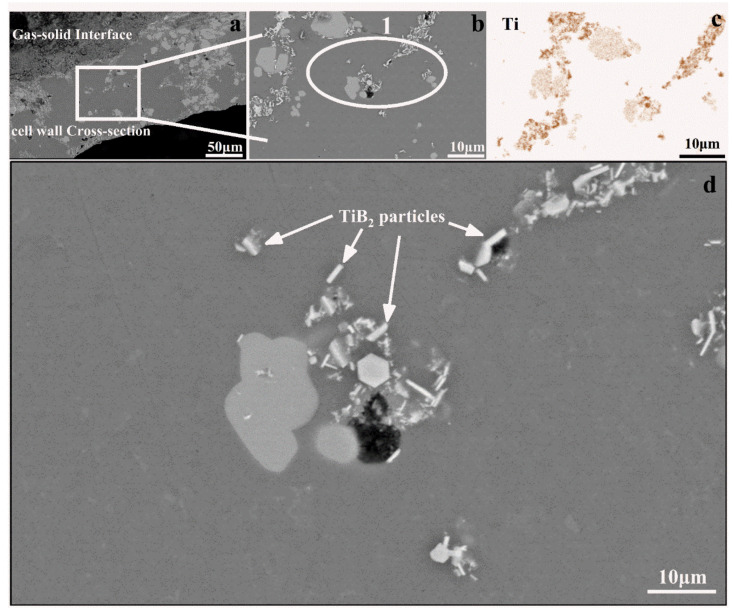
SEM images of Al-5TiB_2_ composite foams: (**a**) cell wall cross-section; (**b**) a magnified view of the cell wall; (**c**) the corresponding Ti elemental mapping in b and (**d**) is a magnified view of the region marked by Circle 1.

The representative microstructures of the Al-5TiB_2_ composite foams are shown in Figure 4. It can be clearly observed that the Ti phases are present in the cell wall cross-section from the Ti elemental mapping (Figure 4c). The Ti phases represent TiH_2_ and TiB_2_. They can be easily distinguished since they are very different in size (16 μm and 0.9 μm, respectively) [18]. Here, TiB_2_ particles are more clearly visible, as shown in Figure 4d. Most of the TiB_2_ particles appear in the form of agglomerates at the cell wall cross-section. It can be determined that the TiB_2_ particles have a faceted morphology and that the average particle size is 1.2 μm, based on the measurement of the longest dimension of about 120 particles.

**Figure 5:**
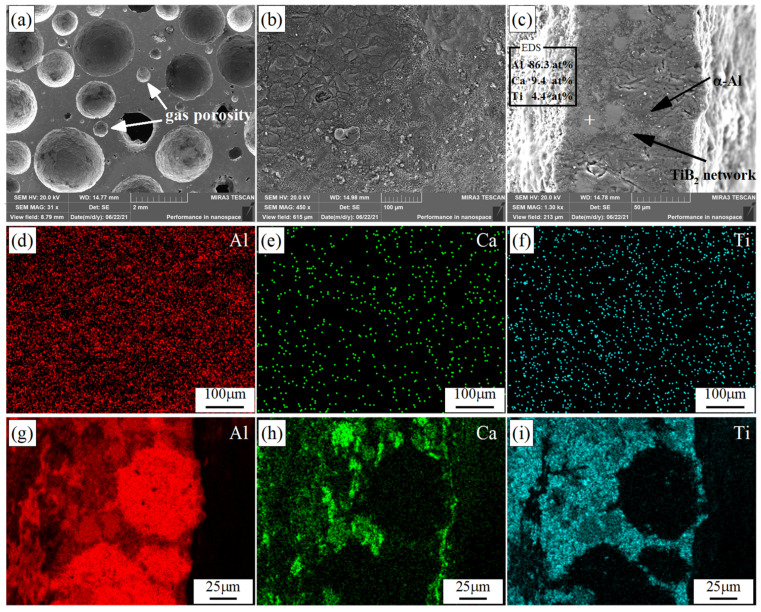
SEM images of the10TiB_2_-0.4P sample: (**a**) macro morphology; (**b**) showing gas-metal interface; (**c**) showing Al-Ca-Ti intermetallic compound and TiB_2_ network; (**d**) Al, (**e**) Ca, (**f**) Ti elemental mappings of image (**b**); (**g**) Al, (**h**) Ca, (**i**) Ti elemental mappings of image (**c**).

SEM images of the 10TiB_2_-0.4P sample are shown in Figure 5. Figure 5a reveals the macro morphology of the 10TiB_2_-0.4P sample. Large gas porosities are seen in the cell walls. The gas pores created by gas release are characterized by regularly shaped pores (usually circular) [14]. Figure 5b reveals the microstructure of the gas-metal interface. Figure 5d–f shows the Al, Ca, and Ti elemental mappings of Figure 5b, respectively. The Ti elemental mapping shown in Figure 5f indicates that the TiB_2_ particles are uniformly distributed on the gas-metal interface. Figure 5c reveals the microstructure of the cell walls showing α-Al grains, Al-Ca-Ti intermetallic compound, and TiB_2_ particles located between the α-Al grains and the intermetallic compound. Figure 5g–i show the Al, Ca, and Ti elemental mappings from Figure 5c, respectively. From the Ti elemental mapping shown in Figure 5f,i, it can be clearly seen that the TiB_2_ particles are present in the inter-dendritic regions and around the Al-Ca-Ti intermetallic compound. Grain refinement of α-Al dendrites can be clearly seen from the microstructures due to the presence of TiB_2_ particles.

**Figure 6:**
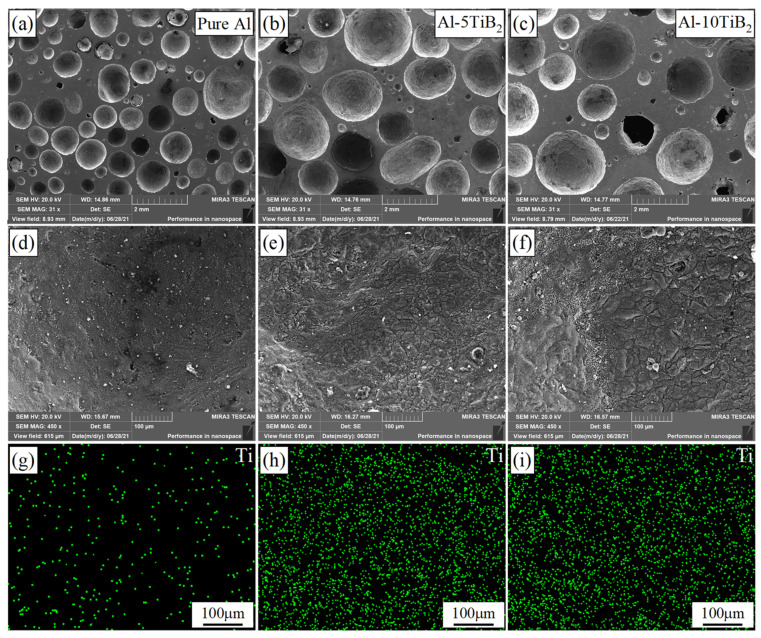
Ti elemental mappings of three different matrix compositions (pure Al, Al-5TiB_2_, and Al-10TiB_2_) in the gas–metal interface: (**a**–**c**) macro morphology and (**d**–**f**) gas–metal interface of (**a**–**c**), respectively; (**g**–**i**) Ti elemental mappings of (**d**–**f**), respectively.

Figure 6 compares the pure Al foam (without TiB_2_) with the Al-TiB_2_ foams fabricated under the same conditions (0.4 MPa). Figure 6a–c shows the macro morphology of pure Al, Al-5TiB_2_, and Al-10TiB_2_, respectively. It can be seen that the average cell size of the Al-TiB_2_ foams is significantly larger than that of pure Al foam. Figure 6d–f show the micro morphology of the gas-metal interface of pure Al, Al-5TiB_2_, and Al-10TiB_2_, respectively. Figure 6g–i show the Ti elemental mappings of (d)–(f), respectively. From the Ti elemental mappings, it can be seen that the Ti content of the pure Al foam is significantly less than that of the Al-TiB_2_ foams, which indicates that the TiB_2_ particles are uniformly distributed on the cell walls of the Al-TiB_2_ foams.

### 3.3. Deformation Initiation

**Figure 7:**
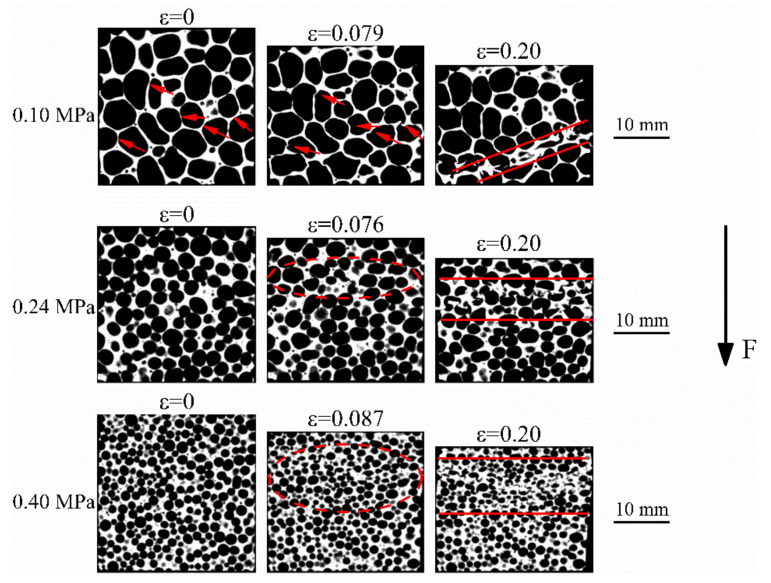
Deformation process of Al-5TiB_2_ foams fabricated under different pressures.

**Figure 8:**
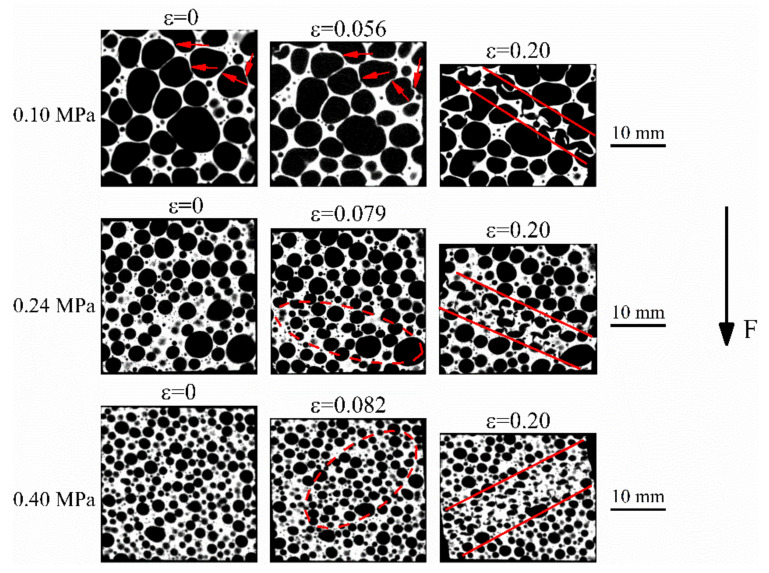
Deformation process of Al-10TiB_2_ foams fabricated under different pressures.

Tomographs obtained from the 5TiB_2_ and 10TiB_2_ foams that were deformed with up to 8% (or 6%) and 20% strain are shown in Figure 7 and Figure 8, respectively. At 8% (or 6%) strain, the cell walls of the 5TiB_2_ (or 10TiB_2_) plastically buckle perpendicular to the load direction, and some cell walls produce slight buckling parallel to the load direction, as shown by the arrows and ellipse zones. However, in the case of 10TiB_2_-0.24P and 10TiB_2_-0.4P, the plastic deformation is not obvious. At 20% strain, the deformation band (the zone between the two red lines) is a mixture of both ductile and brittle in nature in the case of 5TiB_2_-0.1P since both buckling and cracking of thin cell walls can be observed. However, for 10TiB_2_-0.1P, the obvious cracking of the cell walls can be observed in the deformation band, which is consistent with the report that the cell wall of 10TiB_2_ foams is highly brittle in nature [16]. For 5TiB_2_-0.1P and 10TiB_2_-0.1P, the site of the onset of local deformation usually initiates at larger cells generating a concentration of stress in adjacent areas [21,22]. Nevertheless, 10TiB_2_-0.24P and 10TiB_2_-0.4P foams form deformation bands at 45 degrees to the compression direction. The 5TiB_2_-0.24P and 5TiB_2_-0.4P foams show approximately horizontal deformation bands. This may be related to the nature of the cell wall material. It is reported that the 10TiB_2_ foams are very brittle in nature during compression, while such brittleness is not so prominent in the 5TiB_2_ composite foams [16,20]. Therefore, 10TiB_2_ foams tend to experience shear deformation.

### 3.4. Compression Behavior

**Figure 9:**
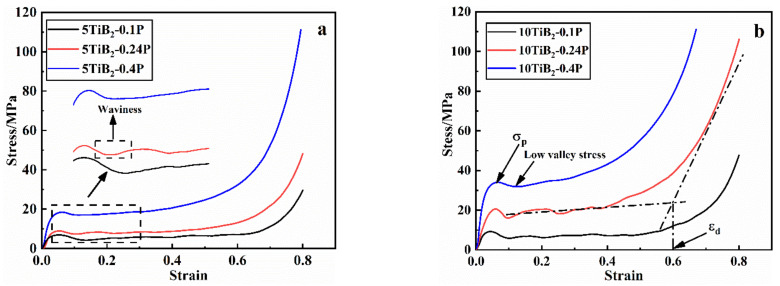
Compressive stress-strain curves of the foams: (**a**) 5TiB_2_ and (**b**) 10TiB_2_.

The typical stress-strain curves of all of the composite foams are shown in Figure 9. All the of the curves show three regions: a linear elastic region at the initial stage, a plateau region with nearly constant flow stress, and a densification region where the stress rapidly increases. The waviness of the stress-strain curve becomes smooth as the cell size is refined. The first peak value in the stress-strain curve was chosen as the yield strength *σ_p_*. *R_sd_* was defined as the stress drop ratio [(difference of *σ_p_* and low valley stress)/*σ_p_*] [6]. The densification strain ε_d_ was determined by finding the intersection point of two lines. The first one was the linear fit to the plateau region, and the second one was the tangent to the densification region, as explained in Ref. [14]. Energy absorption *W* is the area under the stress-strain curve up to *ε_d_* and is calculated by following equation [23]:

(1) $$W\;(\varepsilon d)=\int_{0}^{\;\;\;\;\varepsilon_{d}}\sigma \left(\varepsilon \right)d\varepsilon$$

The yield strength (*σ_p_*) of metal foams is directly related to the density and the strength of the solid constituent. For this, the *σ_p_* values of all of the foams were normalized by the (*ρ_r_*)*^n^* in order to eliminate the effect of density. The exponent n was selected as 1.5 and 2 according to Refs. [14,23]. The corresponding normalized values and compressive properties such as *σ_p_*, *R_sd_*, *ε_d_*, and *W* are given in Table 4.

The deformation mode of foams is the main reason that waviness of the stress-strain curves is induced. Observation of the final deformation stages can elucidate this (see Figure 7 and Figure 8). The crushed bands of the 5TiB_2_-0.1P and 10TiB_2_-0.1P foams are formed due to the complete collapse of a layer of cells. The crushed bands of the 5TiB_2_-0.24P and 10TiB_2_-0.24P foams are formed by the simultaneous collapse of three layers of cells. The crushed bands of the 5TiB_2_-0.4P and 10TiB_2_-0.4P foams are formed by the simultaneous collapse of six and five layers of cells, respectively. The collapse of single-layer cell leads to larger stress drop ratio and fluctuations of the stress-strain curves. However, as the cell size is refined, the simultaneous crush of multiple cells decreases the stress drop ratio and smooths the stress-strain curves.

**Figure 10:**
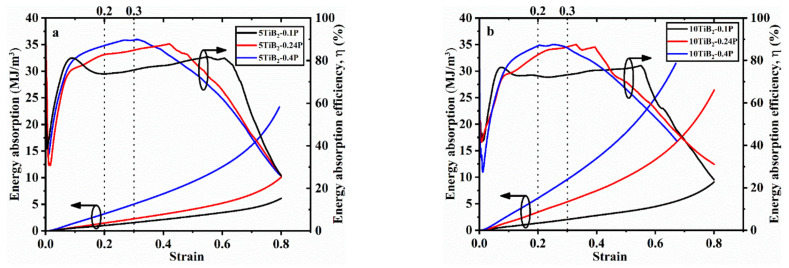
Energy absorption and energy absorption efficiency versus strain of (**a**) 5TiB_2_ and (**b**) 10TiB_2_.

The energy absorption and energy absorption efficiency (η) curves of the foams are shown in Figure 10. *η* is obtained by the following equation [14]:

(2) $$\eta (\varepsilon )=\;\frac{\int_{0}^{\varepsilon }\sigma \left(\varepsilon \right)d\varepsilon }{\sigma_{\max}\left(\varepsilon \right)\varepsilon }$$

where *σ*_max_(*ε*) is the maximum stress up to the strain ε. The *W* and η (maximum value in the curves) are displayed in Table 4. Note that for ideal foams exhibiting a constant plateau stress in compression, *η* = 1 (or 100%), whereas *η* = 0.5 (or 50%) for elastic-brittle solids [24]. As seen in Figure 10, when the foaming pressure increases, the energy absorption of both the 5TiB_2_ and 10TiB_2_ foams increases. This is due to the increase of density, which increases the compressive stress. For the 5TiB_2_-0.1P and 10TiB_2_-0.1P foams, the *η* reaches 0.81 and 0.77 when yielding occurs. Afterwards, it reduces due to the strain softening and reaches 0.82 and 0.78 at the end of the plateau stage. Finally, there is a significant drop due to the compaction of the foams. For the 5TiB_2_-0.24P and 10TiB_2_-0.24P foams, the *η* reaches 0.75 and 0.73 when yielding occurs. Later, it rises to 0.88 and 0.87 and drops significantly during the densification stage. For the 5TiB_2_-0.4P and 10TiB_2_-0.4P foams, the *η* reaches 0.79 and 0.75 when yielding occurs. Later, it rises to 0.90 and 0.88 and drops significantly during the densification stage.

**Figure 11:**
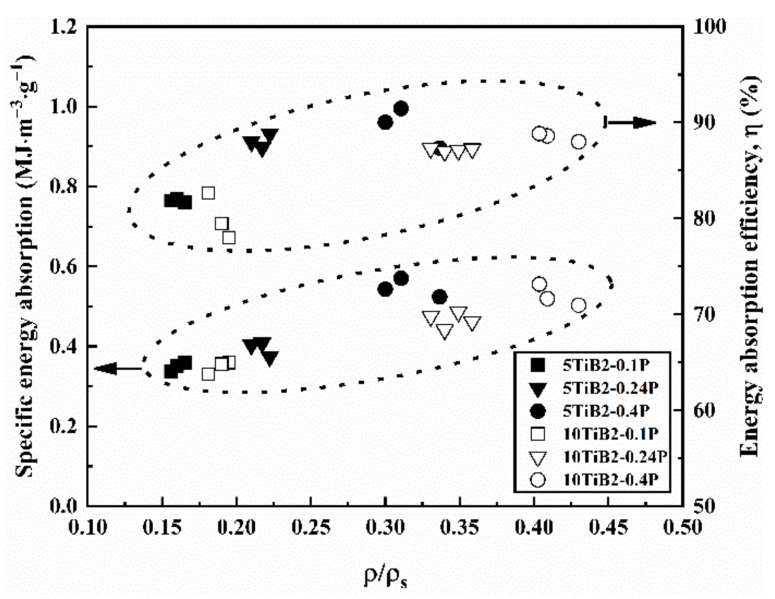
SEA and *η* of foams with different cell structures.

The specific energy absorption (SEA) and *η* versus the relative density of the composite foams with different cell structures are shown in Figure 11. The SEA provides a criterion for the comparison of energy absorbers in their ability to absorb the deformation energy. It can be formulated in several bases, including per unit and volume. The SEA per unit mass is expressed as:

(3) $$\mathrm{SEA}=\frac{E}{m}$$

where m is the structure’s total mass. It can be seen that the SEA and *η* of the composite foams increases when the relative density increases. The energy absorption efficiency is related to the smoothness of the plateau stage in the stress-strain curves. As the pressure increases, a uniform cell structure with fine bubbles is obtained (see Figure 3). This leads to the simultaneous crush of multiple cells (see Figure 7 and Figure 8) and the decrease of fluctuations in the stress-strain curves when pressure increases. As a result, the energy absorption efficiency increases with increasing pressure. In addition, the energy absorption efficiency of the 5TiB_2_ foam is superior to that of the 10TiB_2_ foam, as shown in Figure 11. The ductile and brittle deformation nature for the 5TiB_2_ and 10TiB_2_ foams, respectively, are shown in Figure 7 and Figure 8, which can support this result.

### 3.5. Comparisons

The yield strength of a closed-cell aluminum foam is related to the cell edge bending and cell face stretching. Gibson and Ashby derived an equation for the yield strength σ_p_ of a foam in terms of the foam relative density and properties of the base material [8]:

(4) σpσs=0.3∅32ρρs32+1−∅ρρs

where *σ_s_* and *ρ_s_* are the yield stress and density for the base material, respectively. The term ∅ is the solid fraction that is contained in the cell edges ($\rho /\rho_{s}\leq \emptyset \leq 1$), and the remaining fraction (1−∅) occupies the cell faces.

If the solid fraction in the cell edges is taken as 100%, the foam is open cell, and the equation can be written as follows:

(5) σpσs=0.3ρρs32

Usually, the values of ∅ are between 0.65 and 0.85 [25,26]. However, ∅ is set to 0.75 to compare the experimental results [27,28]. On this basis, the foregoing equation can be written as follows:

(6) σpσs=0.195ρρs32+0.25ρρs

In another study, Simone and Gibson proposed a similar equation using finite element analysis [29,30]:

(7) $$\frac{\sigma_{p}}{\sigma_{s}}=0.33{\left(\frac{\rho }{\rho_{s}}\right)}^{2}+0.44\left(\frac{\rho }{\rho_{s}}\right)$$

**Figure 12:**
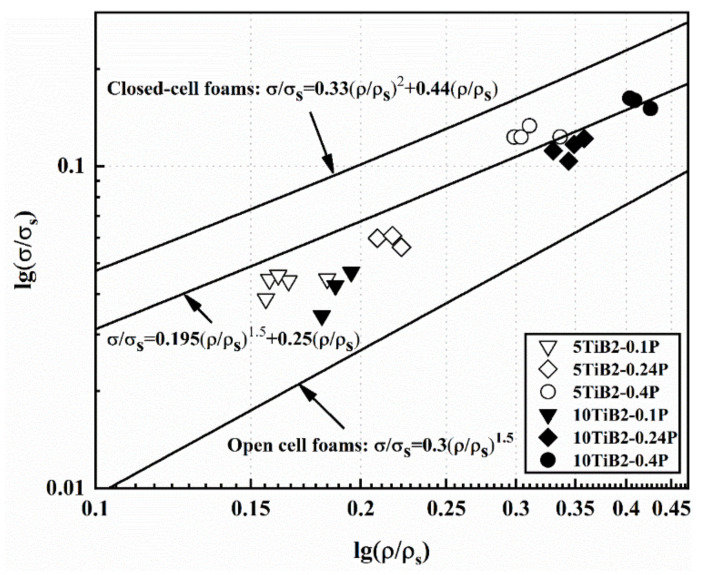
Comparison of experimental data with prediction models.

The comparison of the experimental data with the prediction models is shown in Figure 12. The yield strength of the base material is taken to be the reference value (150 MPa and 200 MPa for 5TiB_2_ and 10TiB_2_, respectively) [16]. The discrete points shown in Figure 12 are the experimental data of the composite foams fabricated under different pressures. It is clear that the strength of the composite foams fabricated under the condition of less 0.20 MPa is slightly lower than the prediction (6). As shown in Equation (4), the contribution from cell edge bending to the overall strength of the foam is by a term that is non-linear in the relative density, while the contribution from cell face stretching is linear in the relative density. In addition, the relative density is much less than 1. Therefore, the influence of cell wall geometry on strength is particularly important. The discrepancy between the experimental data and the prediction model (6) is mainly due to defects, such as cell wall curvature and corrugation, porous inclusions, holes, fractured cell walls, and non-uniform distribution of local density [4,25,27,31]. According to reports, when the ratio (L/2R) of the cell wall length (L) to the curvature radius (R) increases from 0 to 0.5, the strength of closed-cell foams is reduced by 32% to 55% compared to foams with a flat cell wall [32]. However, for composite foams fabricated under the condition of 0.40 MPa, the specific strength is essentially consistent with the prediction model (6). This may be due to the uniform cell structure obtained under increased pressure (see Figure 3), resulting in a reduction in the pressure difference between adjacent cells, which helps to obtain a flat cell wall.

## 4. Conclusions

We have demonstrated that increased pressure is a good strategy for enhancing the mechanical properties of in situ Al-xTiB_2_ (x = 5, 10 wt.%) composite foams through the refinement of the cell structure. It was discovered that the mean cell size decreases, and the cell size distribution narrows with increasing pressure. The study of the stages of deformation revealed that uniform cell structure causes the simultaneous deformation of multi-layer cells, which leads to an improvement in energy absorption efficiency. Moreover, in composite foams fabricated under the condition of 0.40 MPa, the specific strength is mostly consistent with the prediction of G&A model.

## Figures and Tables

**Table 1:** The chemical composition of the Al-10TiB_2_ composite ingot.

Element	Al	Si	Fe	Ti	B	V
wt.%	balance	0.085	0.154	6.835	3.117	0.006

**Table 2:** Foaming parameters.

Al-xTiB_2_	Ca (wt.%)	Stirring Time (min)	TiH_2_ (wt.%)	Stirring Time (min)	Foaming Pressure (MPa)	Foaming Time (min)
x = 5	2.25	5	1.2	3	0.1	10
	2.25	5	1.2	3	0.24	15
	2.25	5	1.2	3	0.4	20
x = 10	2.25	5	1.2	3	0.1	10
	2.25	5	1.2	3	0.24	15
	2.25	5	1.2	3	0.4	20

**Table 3:** Structure properties of 5TiB_2_ and 10TiB_2_ foams. R^2^ indicates the goodness of a fit of the cell size distribution with a log-normal function.

Sample	*ρ* [g/cm^3^]	*ρ_r_*	*D_mean_* [mm]	*R* ^2^	*C_mean_* [Arithmetic Mean]
5TiB_2_-0.1P	0.44	0.16	5.03 ± 0.09	0.81	0.89 ± 0.10
5TiB_2_-0.24P	0.57	0.21	2.87 ± 0.06	0.85	0.90 ± 0.10
5TiB_2_-0.4P	0.81	0.30	1.50 ± 0.02	0.95	0.94 ± 0.13
10TiB_2_-0.1P	0.54	0.19	5.49 ± 0.15	0.64	0.94 ± 0.06
10TiB_2_-0.24P	0.96	0.34	2.99 ± 0.06	0.90	0.94 ± 0.04
10TiB_2_-0.4P	1.19	0.43	1.81 ± 0.06	0.89	0.99 ± 0.07

**Table 4:** Compressive properties of the 5TiB_2_ and 10TiB_2_ foams.

Sample	*σ_p_* [MPa]	σ_p_/(*ρ_r_)^n^* [MPa]		*R_sd_* [%]	*ε_d_* [%]	*W* [MJ m^−3^]	*η* [%]
		*n* = 1.5	*n* = 2				
5TiB_2_-0.1P	6.86	112	280	39.0	69.0	4.17	82
5TiB_2_-0.24P	9.06	105	234	17.9	66.6	6.21	88
5TiB_2_-0.4P	18.47	113	207	8.2	59.5	11.88	90
10TiB_2_-0.1P	9.78	118	271	40.2	66.0	5.28	78
10TiB_2_-0.24P	21.22	107	184	25.3	54.8	11.45	87
10TiB_2_-0.4P	34.57	123	187	8.9	45.7	16.17	88

## Data Availability

The data that support the findings of this study are available from the corresponding author upon reasonable request.
